# Cell-Type Deconvolution of Equine BALF RNA-Seq: A Critical Comparison with Matched Single-Cell Data

**DOI:** 10.3390/genes17070773

**Published:** 2026-06-30

**Authors:** Vidhya Jagannathan, Tosso Leeb, Vinzenz Gerber, Sophie E. Sage

**Affiliations:** 1 Institute of Genetics, Vetsuisse Faculty, University of Bern, 3012 Bern, Switzerland; tosso.leeb@unibe.ch; 2 Next Generation Sequencing Platform, University of Bern, 3012 Bern, Switzerland; 3 Swiss Institute of Equine Medicine, Department of Clinical Veterinary Medicine, Vetsuisse Faculty, University of Bern, 3012 Bern, Switzerland; vinzenz.gerber@unibe.ch

**Keywords:** equine asthma, bronchoalveolar lavage fluid, bulk RNA sequencing, single-cell RNA sequencing, computational deconvolution

## Abstract

**Background/Objectives:** Bulk RNA sequencing (RNA-seq) averages signals across heterogeneous cell populations. Computational deconvolution methods aim to infer cell type composition and cell type-specific gene expression from bulk data, but their performance in equine samples has not been evaluated. In this study, we assessed the ability of computational deconvolution to recover cellular composition and differential expression signals in bronchoalveolar lavage fluid (BALF) from horses with severe equine asthma (SEA) and controls (CTL). **Methods:** Cryopreserved BALF samples from six SEA and five CTL horses previously analyzed by scRNA-seq were used to generate bulk RNA-seq data. The matched scRNA-seq dataset served as the reference for deconvolution. Performance was evaluated by comparing deconvolution raw and mRNA-corrected estimates with scRNA-seq cell proportions. Differential expression between SEA and CTL was analyzed on bulk RNA-seq, deconvoluted expression profiles, and scRNA-seq pseudobulk data. **Results:** Deconvolution primarily captured mRNA-derived cell type proportions rather than true cell counts: agreement with scRNA-seq cell counts was moderate (r = 0.62; 95% CI 0.45–0.75) but improved after mRNA content correction (r = 0.83; 95% CI 0.74–0.89). Comparison with mRNA-weighted scRNA-seq proportions showed near-perfect concordance (r = 0.98; 95% CI 0.97–0.99). Cell type-specific performance varied, with stronger correlations for B cells and dendritic cells and weaker performance for neutrophils, T cells and monocytes/macrophages. Recovery of cell type-specific differential expression was inconsistent, frequently showing cross-lineage signal spillover. Although both approaches detected a Th17 signature in SEA, most deconvolution-derived differentially expressed genes overlapped with conventional bulk RNA-seq results. **Conclusions:** Deconvolution of bulk RNA-seq did not reliably estimate cell counts or provide substantial biological insight beyond conventional bulk analysis, highlighting the value of scRNA-seq for resolving cell type-specific disease mechanisms in equine asthma.

## 1. Introduction

Bulk RNA sequencing (RNA-seq) has long been a cornerstone of transcriptomic analysis, providing valuable insights into gene expression across a wide range of biological contexts. However, one fundamental limitation of bulk RNA-seq lies in its inability to resolve cellular heterogeneity within complex tissues. This challenge is particularly relevant in bronchoalveolar lavage fluid (BALF), whose cellular composition can change markedly in disease states, for example, in equine asthma, one of the most prevalent and clinically significant respiratory disorders in horses [1]. Bulk measurements reflect an average signal across all cells, which can obscure cell type-specific transcriptional profiles and potentially introduce significant biases, a phenomenon referred to as Simpson’s paradox [2]. Single-cell RNA sequencing (scRNA-seq) has emerged as a powerful solution to this problem, enabling the characterization of transcriptomes at single-cell resolution and thereby overcoming the averaging limitations inherent to bulk approaches. In equine respiratory research, scRNA-seq has recently been applied to BALF to investigate severe equine asthma (SEA) [3] and mild equine asthma (MEA) [4]. These studies have provided novel insights into a field that has long struggled to elucidate the underlying immune mechanisms of equine asthma. Notably, they identified a type 3 immune component in SEA [3] and generated new hypotheses [3,4] for further targeted investigation.

Despite its advantages, scRNA-seq remains technically demanding, requiring specialized expertise and dedicated laboratory infrastructure. Importantly, fresh or high-quality viable samples are often necessary, limiting the feasibility of scRNA-seq in field settings and precluding its application to archived specimens. A substantial number of bulk RNA-seq datasets already exist from previous equine studies, representing a potentially rich but underutilized resource. From a 3Rs perspective (Replacement, Reduction, Refinement), it would be highly desirable to leverage these existing data rather than subject new animals to additional sampling.

Recent methodological advances have focused on “resurrecting” bulk RNA-seq datasets by reanalyzing them in the light of cell type-specific transcriptomic signatures derived from scRNA-seq [5]. Computational deconvolution algorithms can infer the cellular composition of bulk RNA-seq samples by comparing their expression profiles to reference signatures of individual cell types. Put simply, scRNA-seq provides the transcriptomic signature of each cell type (for example, upregulation of *TCF4* and *MS4A1* in B cells in equine BALF), and deconvolution algorithms use this knowledge like a codebook to sort the mixed signals in bulk RNA-seq data back into their original cell types. In our example, this approach can identify B cells in the BALF sample, estimate their proportion, and enable comparisons of B cell-specific gene expression between groups (here between asthmatic and control horses). In essence, scRNA-seq has cracked the code, providing a key to unlock the cell type-specific information hidden within bulk RNA-seq datasets.

Before application to experimental bulk RNA-seq datasets, deconvolution algorithms require rigorous validation. To date, most validation efforts have relied on synthetic tissues, i.e., bulk RNA-seq profiles generated in silico, which is considered a silver standard [6]. Although such approaches are informative, the gold standard for evaluating deconvolution performance is the use of paired bulk and single-cell RNA-seq data derived from the same biological samples [7,8,9]. This strategy, which we applied in the present study, enables a direct comparison between estimated and true cellular compositions.

In this study, we sought to evaluate whether computational deconvolution of bulk RNA-seq data is feasible and reliable for equine bronchoalveolar cells. Specifically, we performed bulk RNA sequencing on the same cryopreserved samples for which scRNA-seq data were already available. We hypothesized that deconvolution would enable accurate estimation of the cell type proportions present in BALF samples and that differentially expressed gene (DEG) analyses derived from deconvoluted bulk data would yield biological interpretations comparable to those obtained through scRNA-seq.

## 2. Materials and Methods

**Figure 1:**
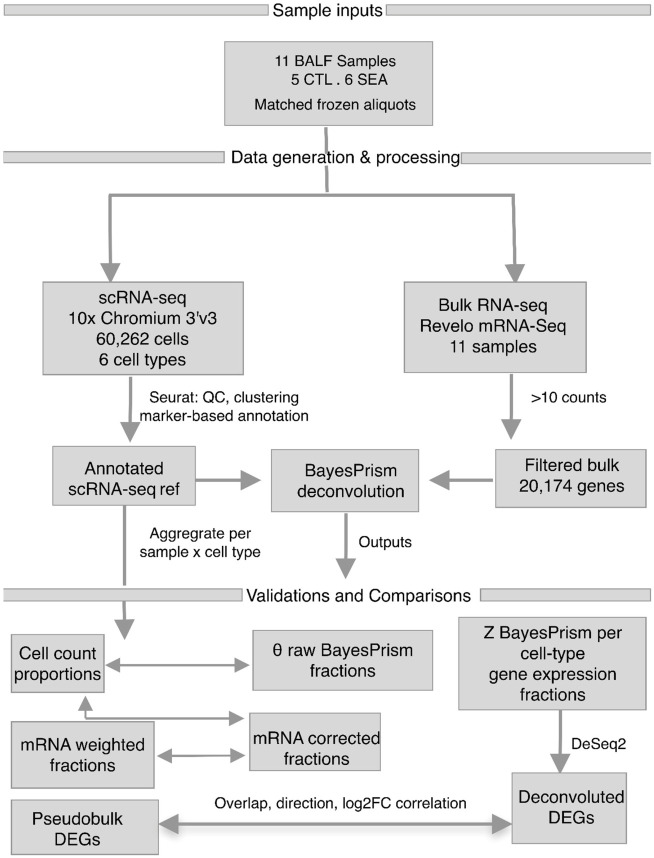
Study design and analytical workflow for matched bulk RNA-seq deconvolution and scRNA-seq-based comparison. Double-headed arrows indicate direct comparisons between analytical outputs. Abbreviations: BALF—bronchoalveolar lavage fluid; CTL—control; SEA—severe equine asthma; DEGs—differentially expressed genes; QC—quality control; log2FC—log2 fold change; ref—reference; RNA-seq—RNA sequencing, scRNA-seq—single-cell mRNA sequencing.

The study design and analytical workflow are presented in Figure 1.

### 2.1. Study Population

Horses were consecutively enrolled from May to December 2020. The present study analyzed 11 BALF samples, comprising six horses with SEA and five control (CTL) horses previously included in a scRNA-seq experiment [3]. Detailed descriptions of inclusion criteria, case selection, study population characteristics, and protocols for sample collection and processing are provided in the supplemental material of our prior scRNA-seq publication [3].

### 2.2. Acquisition of scRNA-Seq Data

Details regarding scRNA-seq data acquisition, library preparation, and sequencing protocols are described in the scRNA-seq publication [3].

### 2.3. Acquisition of Bulk RNA-Seq Data

#### 2.3.1. Thawing and Resuspension of BALF Cell Suspensions

BALF cell suspensions were stored at −80 °C for 4.5 to 5 years prior to RNA extraction. For processing, cryovials containing 500 µL aliquots per horse were rapidly thawed in a 37 °C water bath. Cells were gently resuspended by gradually adding 1 mL of complete growth medium (RPMI supplemented with 10% fetal bovine serum) in five successive steps. Cell suspensions were centrifuged at 300× *g* for 5 min at room temperature. Following removal of the supernatant, 1 mL of medium was retained for cell resuspension. The suspension volume was then brought to approximately 5 mL with additional growth medium, and the centrifugation step was repeated. The supernatant was discarded prior to RNA extraction.

#### 2.3.2. RNA Extraction and cDNA Library Preparation

Total RNA was extracted from cell pellets using the RNeasy Mini Kit (74104, Qiagen, Hilden, Germany) according to the manufacturer’s instructions [10]. RNA was further purified, concentrated, and DNase I–treated using the RNA Clean & Concentrator-5 kit (R1013, Zymo Research, Irvine, CA, USA) following the manufacturer’s protocol. During the RNA normalization step preceding library preparation, a 1% spike-in of Lexogen SIRV-Set 3 RNA controls (Cat. No. 051, Lexogen GmbH, Vienna, Austria) was added to each sample to assess technical variability and sequencing performance. RNA quantity and quality were evaluated using a Qubit 4.0 Fluorometer with the Qubit RNA BR & HS Assay Kit (Q10211 & Q32855, Thermo Fisher Scientific, Waltham, MA, USA). RNA integrity was further assessed on an Advanced Analytical Fragment Analyzer System using the Fragment Analyzer RNA Kit (DNF-471, Agilent Technologies, Santa Clara, CA, USA). Purity of the RNA was determined by spectrophotometry with a DeNovix DS-11 FX spectrophotometer/fluorometer (DeNovix, Wilmington, DE, USA). Gene expression libraries were prepared on a Tecan Revelo platform (Tecan Group Ltd., Männedorf, Switzerland).

#### 2.3.3. Bulk RNA Sequencing

Sequencing libraries were generated from 500 ng input RNA using the Revelo mRNA-Seq for MagicPrep NGS kit A (PN 30186621, Tecan Group Ltd., Männedorf, Switzerland) according to the manufacturer’s instructions [11]. Library concentration and size distribution were assessed using a Qubit 4.0 Fluorometer with the dsDNA HS Assay Kit (Q32854, Thermo Fisher Scientific, Waltham, MA, USA) and an Agilent Fragment Analyzer with the HS NGS Fragment Kit (DNF-474, Agilent Technologies, Santa Clara, CA, USA), respectively.

Pooled libraries were sequenced paired-end (300 cycles) on NovaSeq 6000 and NextSeq 1000 platforms (Illumina, San Diego, CA, USA) using SP Reagent Kit v1.5 (20028400) and P2 Reagents v3 (20046813), respectively, according to the manufacturer’s protocols. Libraries were denatured and diluted according to Illumina guidelines (#1000000106351 v04 and #200027171 v02). Final loading concentrations were 300 pM (NovaSeq) and 750 pM (NextSeq).

Sequencing quality was assessed using Illumina Sequencing Analysis Viewer (v2.4.7). Base call files were demultiplexed and converted to FASTQ format using bcl2fastq (v2.20). RNA extraction, library preparation, sequencing, and quality control were performed at the Next Generation Sequencing Platform, University of Bern.

### 2.4. Bulk RNA-Seq Data Processing

Raw count data from bulk RNA-seq experiments were processed in R (v4.4.1; R Foundation for Statistical Computing, Vienna, Austria). Bulk RNA-seq reads were aligned to the EquCab3.0 (GCF_002863925.1) genome assembly using STAR (v2.7.9a) [12]. The aligned reads were quantified with featureCounts (Subread v2.0.3; options -p -B -C, -g gene_id) [13] against the NCBI Annotation Release 103, with 3′-UTRs extended by 2 kb as described previously [3] and with SIRV/ERCC spike-in sequences appended. Spike-in sequences, featureCounts summary rows, and ribosomal RNA annotation rows were removed from the count matrix used for the downstream analysis.

Genes were excluded if they had fewer than 10 counts in total across the 11 samples, as recommended by default in DESeq2 (v1.44.0) [14]. Marker genes used to define the six cell types were not excluded with this initial filtering. Gene identifiers were harmonized between bulk and scRNA-seq datasets by removing the “gene-” prefix from bulk gene symbols, and sample identifiers were harmonized by removing the “A” prefix from bulk sample names. Quality assessment of the filtered data included library size, gene detection rates, zero-inflation metrics, and the proportion of reads attributed to the top 10 most highly expressed genes per sample (Figure S1).

### 2.5. Computational Deconvolution

#### 2.5.1. Single-Cell RNA-Seq Reference Processing

The reference dataset was generated, quality controlled, and annotated in our previous publication [3] independently of and before the bulk RNA-seq analysis presented here; no re-processing or re-annotation was performed for the present study. The reference scRNA-seq dataset was processed with Cell Ranger (v6.0; 10x Genomics, Pleasanton, CA, USA) against the same EquCab3.0 assembly (GCF_002863925.1) and the same 3′-UTR-extended NCBI Annotation Release 103 as for bulk RNA-seq data. Briefly, cells with <200 or >6500 detected genes or >15% mitochondrial reads were excluded, and counts were normalized using Seurat (v4.0) [15]. Six major cell types were annotated by manually reviewing canonical marker expression after unsupervised Louvain clustering; the full marker list and clustering parameters are provided in [3]. The resulting annotated Seurat object (60,262 cells × 32,835 genes) was used as the deconvolution reference. Cell type annotations were extracted from metadata, comprising 6 major cell types: T cells (49.79%), monocytes/macrophages (MoMa, 37.12%), neutrophils (8.54%), mast cells (2.04%), B cells (1.25%), and dendritic cells (1.25%). The count matrix was transposed to cells-by-genes format and processed to remove genes with minimal expression. No marker gene set was selected for deconvolution, as BayesPrism uses the full overlapping transcriptome (20,174 genes).

#### 2.5.2. Cell Type Deconvolution with BayesPrism

Cell type deconvolution was performed using BayesPrism (v2.2.2) [8] in R (v4.4.1; R Foundation for Statistical Computing, Vienna, Austria). Genes expressed in fewer than 3 cells were filtered from the scRNA-seq reference. The filtered scRNA-seq reference was used for deconvolution. A total of 20,174 genes common between the bulk RNA-seq and filtered scRNA-seq reference were retained. The BayesPrism model was constructed using new.prism with count matrix input, outlier cutoff of 0.01, and outlier fraction of 0.1. Deconvolution was performed using run.prism with 4 cores. Final cell type fraction estimates (*θ*) were extracted using get.fraction. The associated coefficient of variation (CV) statistics output by the algorithm were used to assess uncertainty. CV measures the relative dispersion of the posterior distribution by scaling the standard deviation to the mean, where a lower ratio reflects a more precise and stable estimate.

#### 2.5.3. mRNA Content Correction

Bulk RNA-seq measures total mRNA rather than cell counts, leading to potential overestimation of cell types with high mRNA content per cell (e.g., macrophages) and underestimation of cell types with low mRNA content (e.g., T cells). To address this bias, we implemented the two correction approaches described below.

##### Post Hoc Correction of BayesPrism Estimates

**Figure 2:**
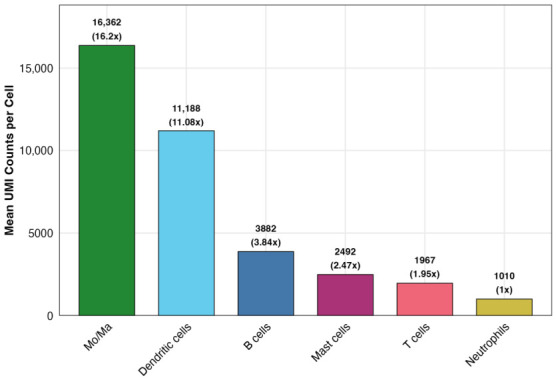
Mean UMI counts per cell across cell types in the experimental scRNA-seq reference dataset. Cell types are ordered by decreasing mRNA content. Values above the bars indicate the mean UMI counts, with the fold difference relative to neutrophils (lowest mRNA content) shown in parentheses. Abbreviations: Mo/Ma—Monocytes/Macrophages; UMI—Unique Molecular Identifier.

The initial fractions (*θ*) estimated by BayesPrism represent the proportion of total mRNA reads contributed by each cell type rather than absolute cell counts. Equating *θ* with cell count proportions ignores cell type differences in per-cell mRNA content, which span ~16-fold in BALF (Figure 2): high-mRNA cell types contribute disproportionately to the bulk signal and are correspondingly overestimated, while low-mRNA cell types are underestimated. To correct for variation in cellular RNA content, we applied a post hoc rescaling similar to the mRNA normalization method used by EPIC [16] and quanTIseq [17]. A multiplicative bias model in which the observed mRNA fraction *θ_k_* of cell type *k* relates to its true cell count fraction *p_k_* as

$$\theta_{k}\;\propto \;p_{k}\cdot m_{k},$$

where *m_k_* is the mean per-cell mRNA content of cell type *k* and *p_k_* is the cell count proportion of cell type *k*. Adjusted estimates are

$${\hat{p}}_{k}\;=\;\frac{\theta_{k}/m_{k}}{\sum_{j}\theta_{j}/m_{j}}.$$

For each cell type, *m_k_* was computed as the mean total UMI count across all reference scRNA-seq cells of that type. The correction assumes that (i) *m_k_* is invariant across samples and disease states, (ii) UMI counts are unbiased proxies for true cellular mRNA content, and (iii) the bias is multiplicative and cell type-specific. Sensitivity of the adjusted estimates was assessed by (i) recomputing *m_k_* under leave-one-sample-out cross-validation of the reference (11 folds) and (ii) recomputing *m_k_* separately for control and asthma samples.

##### mRNA-Weighted Experimental scRNA-Seq

Because deconvolution methods are known to measure mRNA levels rather than actual cell numbers [18], we compared the raw BayesPrism estimates (*θ*) against weighted-mRNA data from the experimental scRNA-seq data. The weighted mRNA data was calculated by summing total UMI counts per cell type within each scRNA-seq sample and normalizing to total library size of the sample. This allows for a direct comparison to raw BayesPrism output (*θ*) and distinguishes true algorithmic error from bias caused by varying mRNA content across cell types.

#### 2.5.4. Condition-Specific Analysis

To assess whether deconvolution accuracy differed between disease states, comparison metrics were also computed separately for CTL (*n* = 5) and SEA (*n* = 6) samples. Per-cell type correlations were calculated within each condition to identify cell type-specific performance differences associated with disease state.

### 2.6. Assessment of Deconvolution Performance

#### 2.6.1. Evaluation of BayesPrism Against scRNA-Seq Reference Data

Deconvolution performance was evaluated by comparing BayesPrism raw estimates against scRNA-seq-derived proportions in matched samples. We computed Pearson correlation coefficients (r) to assess the linear agreement between estimated and observed proportions, Spearman rank correlation (ρ) to assess rank consistency, and mean absolute error (MAE) to quantify the magnitude of deviation. All metrics were calculated across all cell types combined and separately for each individual cell type and per sample. Confidence intervals for both Pearson and Spearman correlations were computed using Fisher’s z-transformation, with the Bonett–Wright standard error correction applied to Spearman correlations [19].

#### 2.6.2. Comparative Analysis Using an Alternative Deconvolution Method (CIBERSORTx)

To test whether the observed mRNA content bias was specific to BayesPrism, we deconvolved the identical bulk RNA-seq mixtures using CIBERSORTx [20]. A custom signature matrix was derived from the same scRNA-seq reference dataset using CPM-normalized expression data and the established cell type phenotype classes. Cell fractions were imputed in relative mode with 100 permutations, quantile normalization disabled, and no batch correction. The resulting CIBERSORTx estimates were evaluated against both the scRNA-seq cell count fractions and the mRNA-weighted proportions using the same statistical metrics applied to BayesPrism (Pearson r, Spearman ρ, mean absolute error).

### 2.7. Concordance of Differential Gene Expression

To assess concordance between cell type-specific DEGs inferred by deconvolution and those derived from the matched scRNA-seq reference, we compared DEGs identified from BayesPrism-deconvoluted bulk RNA-seq against experimental DEGs derived from matched scRNA-seq data.

#### 2.7.1. Pseudobulk Differential Expression from scRNA-Seq

For each of the six major cell types, raw UMI counts were aggregated by sample ID using the AggregateExpression function in Seurat (v5.3.0). Pseudobulk count matrices were constructed with samples as columns and genes as rows.

#### 2.7.2. Extraction of Cell Type-Specific Expression from Deconvolution

Cell type-specific gene expression profiles were extracted from the BayesPrism output using the get.exp function with state.or.type = “type”. This generated the Z matrix, representing the posterior mean of fractional reads attributed to each cell lineage. For differential expression analysis, the matrix was transposed to a gene-by-sample format. The fractional imputed counts were rounded to the nearest integer for DeSeq2. Differential expression analysis was performed on the native BayesPrism Z matrix, as mRNA content correction applies to cell type proportion estimates (*θ*) rather than to cell type-specific expression profiles, which are already decomposed per lineage.

#### 2.7.3. Bulk RNA-Seq Expression Data

For conventional bulk RNA-seq analysis, the filtered count matrix (see Section 4) was used directly as input for differential expression testing between SEA and CTL.

#### 2.7.4. Differential Gene Expression Between SEA and CTL

Differential gene expression between SEA and CTL was analyzed using DESeq2 (v1.44.1) for experimental scRNA-seq, deconvoluted expression data and bulk RNA-seq. Identical parameters were applied to all datasets to ensure comparability. Size factors were estimated using the poscounts method to account for zero-inflated count distributions and genes with fewer than 10 total counts across all samples were excluded. Statistical testing was performed using a Wald test with CTL as the reference condition. Genes with a Benjamini–Hochberg adjusted *p*-value < 0.05 and an absolute log2 fold change (|log2FC|) > 1 were considered differentially expressed.

#### 2.7.5. DEG Comparison Metrics

Concordance between the experimental scRNA-seq (pseudobulk) and deconvoluted results was assessed using three metrics computed on genes shared between datasets. Log2FC correlation was quantified by Pearson and Spearman coefficients to evaluate the linearity and rank consistency between methods. DEG overlap was determined by identifying significant genes with adjusted *p*-value < 0.05 and |log2FC∣ > 1. Directional concordance was defined as the proportion of genes significant in either method (union of DEG sets) with matching log2FC signs (up- or downregulated) between pseudobulk and deconvolution. This metric evaluates whether genes identified as differentially expressed by at least one method show consistent direction of change across both analytical approaches, regardless of statistical significance in the other method.

Additionally, a three-way comparison was performed to assess the overlap of DEGs identified by conventional bulk RNA-seq, scRNA-seq pseudobulk and deconvolution. For this analysis, significant DEGs (adjusted *p*-value < 0.05 and |log2FC| > 1) were pooled across all cell types for both the pseudobulk and deconvoluted analyses. Pairwise and three-way intersections were computed to quantify the number of genes shared between methods as well as those unique to each approach.

## 3. Results

### 3.1. Bulk RNA-Seq Dataset

Six SEA samples and five CTL samples were subjected to bulk RNA-seq. The median RNA Quality Number across all analyzed samples was 7.9 (range: 7.3–9.3), indicating high RNA integrity. Individual bulk RNA libraries yielded between 18.2 and 101.8 million reads, with 17,293 to 20,665 genes identified per sample (Figure S1). Data sparsity (zero inflation) ranged from 4.9% to 20.4%, while the top 10 most abundant genes accounted for 7.3% to 11.1% of total reads across samples.

### 3.2. mRNA Content Varies Across Cell Types

Analysis of scRNA-seq data revealed substantial differences in mean mRNA content (total UMI counts) across cell types (Figure 2). Neutrophils had the lowest mRNA content (1010 UMI/cell), with T cells also showing relatively low expression levels (1967 UMI/cell; 1.95-fold higher than neutrophils). MoMa showed the highest mean mRNA content (16,362 UMI/cell; 16.2-fold), followed by dendritic cells (11,188 UMI/cell; 11.1-fold).

### 3.3. Baseline Deconvolution

BayesPrism deconvolution of bulk RNA-seq data estimated cell type proportions (*θ*) across all 11 matched samples. MoMa dominated the inferred cellular composition, comprising 61–86% of total proportions (mean 75%) across samples. T cells were estimated at 7–30% (mean 21%), followed by neutrophils at 1–8% (mean 3%). Dendritic cells, mast cells, and B cells were estimated at low proportions (<5%, <2%, and <0.1%, respectively).

Uncertainty in the estimated proportions, quantified by the coefficient of variation (CV), varied substantially across cell types. Estimates for dominant cell types showed low uncertainty, with MoMa displaying the lowest CV (0.01–0.03%), followed by T cells (0.05–0.16%) and neutrophils (0.13–0.78%). In contrast, rarer cell types showed higher uncertainty: mast cells showed moderate variability (CV 0.4–3.6%), whereas B cells (CV 0.8–112%) and dendritic cells (CV 0.2–105%) displayed the greatest variability.

**Figure 3:**
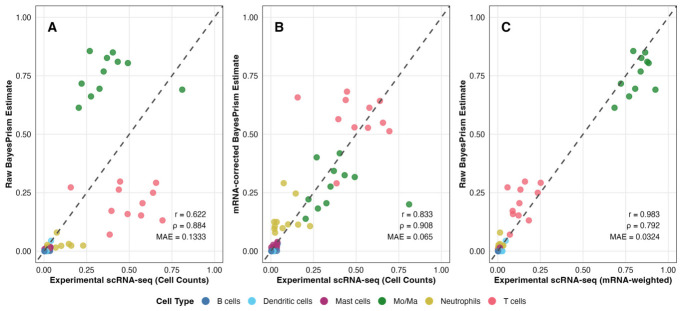
Comparison of deconvolution estimates with experimental scRNA-seq data. Scatter plots comparing deconvolution-estimated cell type proportions with experimental scRNA-seq-derived proportions for matched samples. (**A**) Raw BayesPrism estimates vs. experimental scRNA-seq cell count proportions, showing systematic overestimation of MoMa and underestimation of T cells. (**B**) mRNA-corrected BayesPrism estimates vs. experimental scRNA-seq cell count proportions, demonstrating reduced systematic bias after correction. (**C**) Raw BayesPrism estimates vs. mRNA-weighted experimental scRNA-seq proportions, confirming that deconvolution accurately captures mRNA-derived proportions. Points are colored by cell type; dashed line indicates perfect agreement (*y* = *x*). Abbreviations: MAE—mean absolute error; scRNA-seq—single-cell mRNA sequencing.

Comparison with scRNA-seq-derived cell count proportions yielded an overall Pearson correlation of r = 0.62 and a Spearman correlation of ρ = 0.884 (Figure 3A). Cell type-specific analysis (Table 1) showed that B cells and dendritic cells maintained strong correlations and low MAE. Conversely, MoMa and T cells displayed higher error margins and lower concordance, with an overestimation of MoMa and underestimation of T cells relative to experimental counts.

### 3.4. mRNA-Adjusted Deconvolution

To assess whether the poor correlations for dominant cell types were attributable to mRNA content bias, we implemented a correction for cellular mRNA content (see Methods Section 2.5.3). The correction reduced the compositional bias and improved overall deconvolution accuracy, with correlation increasing from r = 0.62 (95% CI 0.45–0.75) to r = 0.83 (95% CI 0.74–0.89) (Figure 3B, Table 1 and Table S4). In the raw BayesPrism output (*θ*), MoMa dominated at 61–86% across samples. Following mRNA content correction, MoMa proportions decreased to 14–42%, while T cells increased from 7–30% to 29–68% and neutrophils from 1–8% to 8–29%. MAE decreased from 0.40 to 0.13 for MoMa and from 0.31 to 0.15 for T cells, though correlations with experimental scRNA-seq remained poor for both populations (r < 0.2). B cells and mast cells showed improved correlations following correction (r = 0.90 and 0.67, respectively) with consistently low error. Neutrophils MAE increased from 0.06 to 0.09 following correction. Sensitivity analyses confirmed that the corrected estimates were robust to sample-level variation in the scRNA-seq reference (leave-one-sample-out: overall r = 0.821–0.836 across 11 folds) and to disease state-specific scaling (r = 0.830). See Table S6 for full per-cell type *m_k_* values across all folds and conditions.

### 3.5. Comparison with mRNA-Weighted Experimental scRNA-Seq

**Figure 4:**
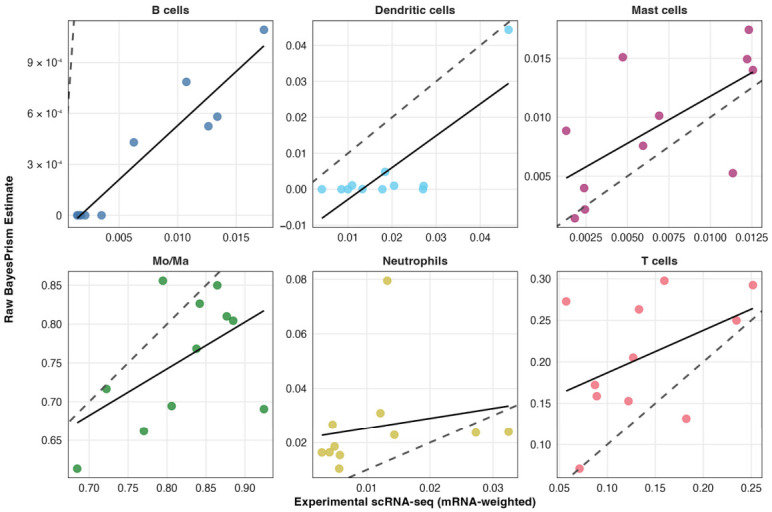
Cell type-specific concordance between BayesPrism estimates and mRNA-weighted experimental scRNA-seq. Scatter plots compare the estimated fraction (*θ*, *y*-axis) against the mRNA-weighted experimental scRNA-seq (*x*-axis) for each of the six major cell types. Each point represents a biological sample (*n* = 11). The solid black line indicates the linear regression fit (Pearson correlation trend), while the dashed line represents the line of identity (*y* = *x*), indicating perfect prediction. B cells and dendritic cells exhibit strong linear alignment, whereas neutrophils show limited concordance consistent with low transcriptional signal. Abbreviations: MoMa—Monocytes/Macrophages; scRNA-seq—single-cell mRNA sequencing.

Comparing BayesPrism estimates (*θ*) against mRNA-weighted experimental scRNA-seq data (see Methods Section 2.6.1) showed near-perfect global concordance (r = 0.98; 95% CI 0.97–0.99; MAE = 0.003) across all samples (Figure 3C, Table 1 and Table S4). The mRNA-weighted scRNA-seq data was dominated by MoMa across all samples (69–92%), in contrast to cell count-based proportions where T cells were predominant (Figure S2). At the individual cell type level (Figure 4 and Table 1), concordance was highest for B cells and dendritic cells, moderate for mast cells, and weaker for MoMa and T cells. Neutrophils showed the lowest agreement with mRNA-weighted scRNA-seq proportions.

### 3.6. Deconvolution by Condition (CTL vs. SEA)

Comparison of deconvolution accuracy between CTL (*n* = 5) and SEA (*n* = 6) samples revealed condition-dependent performance differences (Figure S3 and Table 2). Overall Pearson correlations between raw deconvolution estimates and experimental scRNA-seq cell counts were similar across conditions (Table 2 and Table S5). However, at the cell type level, concordance was markedly higher in SEA than in CTL samples (Figure S3 and Table S1), with the largest differences observed in B cells and T cells.

### 3.7. Cell Type Composition by Condition

**Figure 5:**
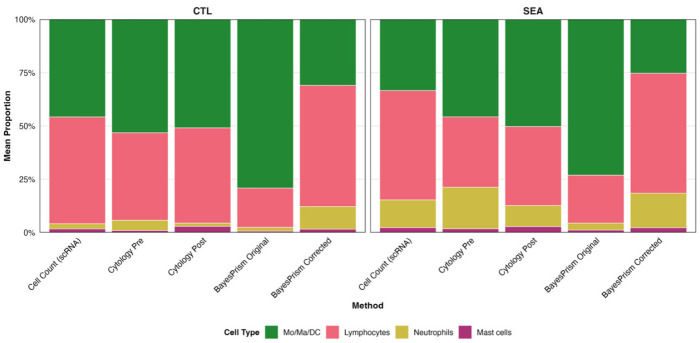
Cell type composition by condition and method. Cell types were merged to match cytology classification (MoMa with dendritic cells; lymphocytes combining T and B cells). Abbreviations: Cytology Pre—cytology before BALF processing; Cytology Post—cytology after BALF processing (before freezing); DC, dendritic cells; MoMa—Monocytes/Macrophages; scRNA—single-cell mRNA sequencing; CTL—control; SEA—severe equine asthma.

Mean cell type composition was compared across methods and stratified by condition (CTL vs. SEA) (Figure 5). Cell types were merged to match cytology classification: lymphocytes (T cells + B cells) and MoMa/DC (Monocytes/Macrophages + Dendritic cells) (Table S2). Across both conditions, the raw BayesPrism output (*θ*) showed overestimation of the MoMa/DC fraction (73.1–79.2%) and a corresponding underestimation of lymphocytes (18.4–22.5%) relative to scRNA-seq and cytology data. Following mRNA content correction of BayesPrism estimates, the compositions aligned more closely with cytology and scRNA-seq cell proportions. In the CTL group, corrected MoMa/DC proportions decreased to 31.0%, while lymphocytes increased to 56.8%, compared to scRNA-seq counts (45.8% and 50.1%, respectively). In the SEA group, the correction reduced MoMa/DC estimates from 73.1% to 25.3%, and elevated lymphocytes from 22.5% to 56.3%. The corrected estimates for neutrophils (10.6–16.2%) were higher than scRNA-seq (2.4–13.0%) values in both conditions.

### 3.8. Robustness Check Using CIBERSORTx

To confirm that these findings were not an artifact of the BayesPrism framework, we analyzed the same bulk RNA-seq samples using a second independent deconvolution method, CIBERSORTx, which utilizes support vector regression. Consistent with the BayesPrism results, CIBERSORTx demonstrated a high correlation with the mRNA-weighted scRNA-seq proportions (r = 0.98) but correlated poorly with the cell count fractions r = 0.44, ρ = 0.42, MAE = 0.20; Figure S4). Crucially, the direction of the estimation error matched that of BayesPrism: MoMa (which exhibit high per-cell mRNA content) were substantially overestimated (mean of 0.95 vs. 0.38 by cell count), while T cells (low per-cell mRNA content) were almost entirely missed (0.01 vs. 0.50 by cell count; Figure S4).

### 3.9. Differential Gene Expression Analysis: Deconvolution vs. scRNA-Seq

- Cell type-specific DEGs: number and overlap

To assess whether BayesPrism deconvolution accurately recovers cell type-specific transcriptional changes associated with SEA, we compared DEGs identified from deconvoluted bulk RNA-seq data against DEGs derived from matched scRNA-seq analysis. All six major cell types were analyzed. DESeq2 analysis was performed on experimental scRNA-seq (pseudobulk) and deconvoluted expression matrices for each cell type, comparing SEA with CTL (Table 3). Pseudobulk analysis detected substantially more DEGs in neutrophils (1195) and T cells (106) than deconvolution (19 and 84, respectively), whereas MoMa showed the opposite pattern with more DEGs detected by deconvolution (93 vs. 33). Mast cells and dendritic cells showed minimal differential expression with either method. B cells showed 57 DEGs with pseudobulk and none with deconvolution. This reflects the absence of detectable B cell signals in CTL samples after deconvolution, likely due to their low abundance (86 vs. 670 B cells in SEA, ~eightfold lower).

b. DEG concordance

**Figure 6:**
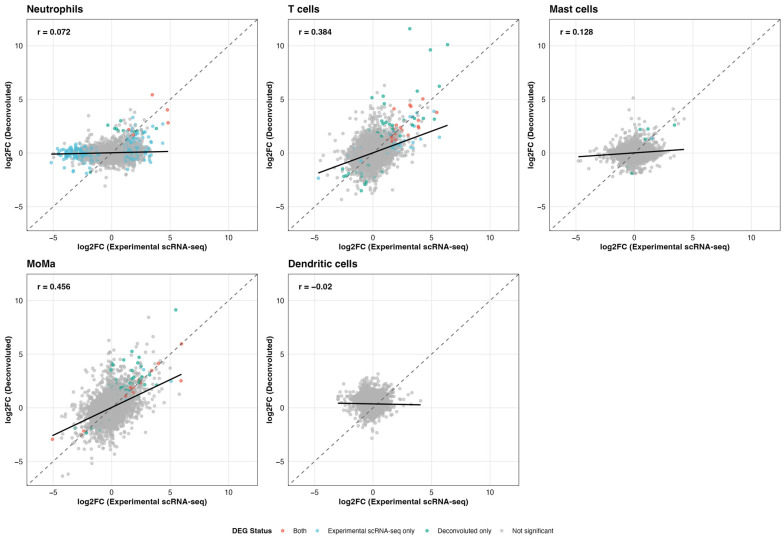
Log2 fold change correlation between experimental scRNA-seq (pseudobulk) and BayesPrism deconvolution-derived differential expression across cell types. Each point represents a gene shared between both datasets. The dashed line indicates perfect agreement (slope = 1) and the solid line shows the linear regression fit. Pearson correlation coefficients (r) are displayed per panel. Points are colored by significance status: red—significant in both methods; blue—significant in experimental scRNA-seq only; green—significant in deconvolution only; gray—not significant in either (adjusted *p*-value < 0.05 and |log2FC| > 1). Abbreviations: DEG—differentially expressed gene; log2FC—log2 fold change; MoMa—Monocytes/Macrophages; scRNA—single-cell mRNA sequencing.

The accuracy of deconvolution-derived DEGs was quantified by comparing them to pseudobulk using genes common to both datasets (Table 4 and Figure 6). Pearson correlations of log2FC estimates showed that MoMa and T cells had the strongest agreement (r = 0.456 and r = 0.384, respectively), whereas neutrophils, mast cells, and dendritic cells displayed weak correlations (r < 0.13). Direction concordance—defined as the proportion of genes significant in either method that showed matching fold change direction (see Section 2.7)—was highest for MoMa (95.9%) and T cells (94.3%). This indicates that even when statistical significance differed between methods, the direction of expression change was largely consistent (Table 4). Neutrophils showed direction concordance of only 51.2%, barely exceeding random chance. Mast cells exhibited 100% direction concordance despite a lack of shared significant genes (reflecting the fact that concordance was calculated across all genes with available log2FC estimates rather than restricted to overlapping DEGs). Spearman rank correlations of log2FC followed a similar pattern, with MoMa (ρ = 0.409) and T cells (ρ = 0.300) showing moderate agreement, while neutrophils showed no rank consistency (ρ = −0.029).

c. Biological interpretation of differential gene expression

T cells

Both BayesPrism deconvolution and scRNA-seq pseudobulk analysis identified a dominant Th17 inflammatory signature in T cells from SEA horses, with concordant upregulation of *IL17A*, *IL26* and *CCL20*. This finding is consistent with previous analysis of the same dataset using NEBULA [3,21] and with flow cytometry demonstrating increased IL-17A-producing lymphocytes in BALF from SEA horses following cell stimulation [22]. Both approaches also detected increased expression of *TOX2*, suggesting T cell exhaustion [23], as well as regulators of T cell–B cell interactions including *CTLA4* and *ICOS* [24,25].

Method-specific signals were also observed. Deconvolution uniquely identified upregulated *IL21*, a regulator of Th17 maintenance and germinal center B cell responses [26]. In contrast, pseudobulk analysis identified additional Th17-associated genes not detected by deconvolution, including *IL17F*. It also revealed upregulation of Vitamin D Receptor gene *VDR*, suggesting a potential compensatory anti-inflammatory response [27], and *ITGB8*, associated with airway remodeling through TGF-β activation [28].

B cells

Pseudobulk analysis identified 57 DEGs in B cells from SEA horses, whereas deconvolution detected none. The pseudobulk DEG profile was dominated by canonical B cell lineage and B cell receptor genes (*POU2AF1*, *EBF1*, *IKZF3*, *CD19*, *CD79A*) as well as genes involved in antigen presentation and interferon-associated processing (*HLA-DRA*, *PSMB8*, *IRF1*). Several genes involved in redox regulation and oxidative stress responses (*PRDX4*, *GPX1*, *ALDH2*) were also upregulated, suggesting adaptation of B cells to an oxidatively stressed airway environment. Only three genes were downregulated including *LGALS1* (galectin-1), which inhibits Th17 differentiation and promotes Th17 cell apoptosis [29]; its reduced expression may therefore favor Th17-driven inflammation.

Monocytes/Macrophages

Shared DEGs included *FABP7* and *LYZ*, both upregulated in SEA. *FABP7* has been implicated in PPARγ-mediated M2 polarization and profibrotic responses [30], while *LYZ* supports antimicrobial macrophage activation. A substantial proportion of deconvolution-derived genes corresponded to markers typically associated with other lineages, including B cells (*PAX5*, *EBF1*), T cells (*IL17A*, *IL26*), and epithelial cells (*MUC4*, *PIGR*).

Pseudobulk-specific DEGs revealed MoMa-intrinsic inflammatory and remodeling programs: *CXCL13* (Th17-associated chemokine mediating B cell recruitment), *CCL14/CCL15* (CCR1 ligands driving monocyte egression to inflamed lung [31], *DEFB1*, *TLR1* (innate antimicrobial defense), and *MMP8* (matrix metalloproteinase involved in airway remodeling [32]).

Dendritic cells

No statistically significant DEGs were detected in dendritic cells using either method.

Neutrophils

Among shared genes, *ECATH-3* (cathelicidin stored in neutrophil granules) supported antimicrobial function [33]. However, several overlapping genes were not neutrophil-restricted, including *FABP7* (macrophage-associated), *IGLC6* (immunoglobulin λ constant), and *TGM3* (keratinocyte-associated transglutaminase), suggesting residual cross-lineage contamination even among shared signals.

Pseudobulk-specific DEGs revealed a broad neutrophil activation program consistent with NET formation: upregulation of *S100A8*, *S100A9*, *RETN* and *PADI4* [34,35,36,37], as well as *CHI3L1* and *MAPK13*, previously associated with neutrophilic asthma in humans [38,39,40]. Deconvolution-exclusive DEGs were limited, with *GFI1B* (a transcription factor implicated in neutrophil differentiation [41]) being the only lineage-relevant finding.

Mast cells

Deconvolution identified seven DEGs in mast cells (five up, two down). One upregulated gene, *NDRG1*, has been linked to mast cell degranulation [42], but the remaining DEGs lacked mast cell specificity, including *LYZ* and *JCHAIN* (markers of myeloid and plasma cell lineages, respectively). Pseudobulk identified a single upregulated gene (*LOC111771745*, a granzyme B-like locus), consistent with activated mast cell secretory function [43], which was not captured by deconvolution.

### 3.10. Deconvolution Versus Bulk RNA-Seq Differential Expression

To assess whether deconvolution provides biological insight beyond conventional bulk analysis, we compared bulk DEG results with cell type-resolved DEGs from BayesPrism (Table 5). Deconvolution identified 203 DEGs across six cell types (130 unique gene symbols after collapsing redundancy). Of these, 100 (76.9%) were shared with the bulk DEG list (133 DEGs), with 100% directional concordance.

The 30 deconvolution-exclusive genes (after collapsing redundancy across cell types) were distributed primarily across T cells and MoMa. However, the added biological value was modest. MoMa-exclusive genes predominantly corresponded to markers of other cell types, indicating cross-lineage contamination. In neutrophils and mast cells, deconvolution-exclusive DEGs had no clear interpretive value. Only the T cell compartment yielded deconvolution-exclusive findings of genuine biological interest with upregulation of *CTLA4* and *ICOS*, both absent from the bulk DEG list.

## 4. Discussion

In this study, we evaluated the performance of BayesPrism for deconvolving bulk RNA-seq data from equine BALF using a matched experimental scRNA-seq reference. Many benchmarking studies rely on pseudobulk simulations, which represent a silver standard rather than a true gold standard [6]. Indeed, in silico simulations do not capture technical variability introduced during sample processing and library preparation, nor biological variability between individuals, such as differences in cell type abundance and gene expression profiles. By using matched bulk and single-cell data from the same samples, we evaluated BayesPrism performance in equine BALF while preserving the technical and biological variability inherent to experimental datasets. The resulting assessment is therefore specific to this tissue and cohort. We found that BayesPrism accurately estimated cell type mRNA contributions (overall r = 0.983) but deviated from cell count-based proportions (overall r = 0.622). This discrepancy was attributed to the >16-fold variation in mRNA content across BALF cell types, as applying a post hoc mRNA correction significantly improved agreement with cell count proportions (overall r = 0.833). Deconvolution-derived cell-type-specific DEGs (SEA vs. CTL) showed moderate concordance with experimental scRNA-seq results for the most abundant cell types. Furthermore, deconvolution did not substantially improve the biological interpretation of differential gene expression compared with conventional bulk RNA-seq analysis.

### 4.1. mRNA Bias Drives Error

MoMa displayed a 16.2-fold higher mean UMI count per cell relative to neutrophils, with dendritic cells showing an 11.1-fold increase. Similar transcriptome size differences have been reported in human immune cells, where monocytes contain more mRNA content than neutrophils and lymphocytes [44]. Uncorrected BayesPrism estimates indicated an average of 75% MoMa across samples, compared with 38% based on experiment scRNA-seq, while T cells were underestimated (21% vs. 50%). Such bias towards high-mRNA cell types is a known limitation of transcriptomic deconvolution [45,46]. Because these methods primarily quantify mRNA fractions rather than cell counts, cell types with higher mRNA content per cell contribute disproportionately to the bulk signal and are therefore overestimated [46]. Our results in equine BALF provide empirical support for this observation. CIBERSORTx yielded similar patterns of error, overestimating MoMa still further (mean of 95%) and almost entirely missing the T cell population (1%). Because both independent algorithmic frameworks suffer from the same directional error, these findings indicate that the bias toward estimating mRNA-derived cell proportions rather than true cell proportions is an intrinsic property of reference-based deconvolution rather than an artifact of any single software package.

### 4.2. Correction Reduces Estimation Bias

A post hoc correction was applied by dividing BayesPrism estimates by the mean mRNA content per cell type and renormalizing. This improved the overall Pearson correlation from 0.622 to 0.833 and halved the overall MAE from 0.133 to 0.065. This approach is conceptually similar to mRNA normalization strategies implemented in EPIC [16] and quanTIseq [17], which adjust estimated mRNA fractions using cell type-specific mRNA content to approximate cell count proportions. Similarly, the recently introduced ReDeconv algorithm [46] incorporates transcriptome size directly into its deconvolution model.

However, the correction was not perfect. MoMa estimates decreased from 75% to 28%, indicating slight overcorrection, while lymphocyte proportions increased to 57%. These residual discrepancies likely arise from the simplifications inherent to the uniform scaling approach: dividing by a single mean mRNA value per cell type assumes constant transcriptional output across activation states and disease conditions, an assumption that may not hold for immune cells in inflamed airways. Moreover, because corrected fractions are renormalized to sum to one, overcorrection in one cell type inevitably skews estimates of others. Nonetheless, the corrected proportions more closely matched both cytology counts and scRNA-seq-derived values, demonstrating that post hoc correction can improve the accuracy of relative cell-type abundance estimates.

### 4.3. Cell Type-Specific Deconvolution Performance

Deconvolution accuracy varied across cell types and appeared to depend more on how distinct their gene expression profiles were than on how abundant they were. B cells and dendritic cells showed strong correlations with experimental scRNA-seq (r = 0.87 and 0.90, respectively), despite being relatively rare cell populations. This likely reflects their large sets of unique marker genes (636 and 1033) (Table S3), which provide a high signal-to-noise ratio within the bulk mixture.

Neutrophils were challenging to deconvolve. Their low basal transcriptional activity, short half-life and high endogenous RNase levels make them inherently difficult to profile at the transcriptomic level [47]. Neutrophils had the lowest mRNA content in our dataset (1010 UMI per cell), resulting in a minimal contribution to the bulk RNA-seq signal, and limiting the information available for accurate deconvolution. This observation aligns with previous benchmarking studies showing that rare or low-mRNA cell types are systematically more difficult to deconvolve [48,49].

### 4.4. Condition-Specific Performance Reveals Biological Heterogeneity

Deconvolution performance differed between CTL and SEA samples. Although overall Pearson correlations for the original estimates were similar (0.623 in CTL; 0.625 in SEA), mRNA-corrected estimates performed substantially better in SEA (r = 0.908) than in CTL (r = 0.771). At the cell type level, MoMa and T cells showed strong negative correlations in CTL (r = −0.90 and −0.89) but positive correlations in SEA (r = 0.82 and 0.58). In CTL, these negative correlations were largely driven by a single sample with an atypical composition (81% MoMa, 16% T cells, compared with 27–44% MoMa in the remaining CTL samples), demonstrating that one outlier was sufficient to invert the correlation pattern. These findings highlight the instability of per-cell type correlation estimates in small datasets and caution against overinterpreting condition-specific metrics when sample size is below ten.

### 4.5. Concordance of Cell Type-Specific Differential Expression with the Reference scRNA-Seq Dataset

BayesPrism infers cell type-specific gene expression (Z matrix), allowing for direct comparison of deconvolution-derived DEGs with experimental scRNA-seq results. Moderate concordance was observed for MoMa and T cells, with 55% and 23% of significant DEGs overlapping with the scRNA-seq reference, respectively, direction agreement of 96% and 94% and log2FC Pearson correlations of 0.456 and 0.384.

In contrast, neutrophils showed striking discordance: experimental scRNA-seq identified 1195 DEGs, whereas deconvolution detected only 19, with five overlapping genes. Direction concordance was essentially random (51%), and log2FC correlation was negligible. This likely stems from neutrophils’ low mRNA content and their minimal contribution to the bulk RNA-seq signal, which restricts the information available to infer neutrophil-specific expression changes.

Consistent with Meng et al. [50], our results suggest that while marker distinctiveness drives deconvolution accuracy, DEG recovery depends on a cell type’s total transcriptomic signal, a product of both abundance and per-cell mRNA content. Consequently, the combination of low frequency and minimal mRNA in neutrophils creates a signal too weak for reliable differential expression analysis.

### 4.6. Deconvolution Did Not Significantly Improve Biological Interpretation

Both approaches converged on the identification of a neutrophilic, type-17-skewed inflammatory environment in SEA. However, they differed markedly in resolution and cellular attribution.

Deconvolution captured broad inflammatory signals but incompletely resolved cell type-specific activation programs and frequently redistributed transcriptional signals across cell compartments. In contrast, scRNA-seq pseudobulk preserved cell identity and revealed coordinated transcriptional programs, including NETosis, chemokine-mediated myeloid recruitment, and B cell activation.

Comparison with bulk RNA-seq showed that deconvolution provided limited additional biological insight. Bulk data already captured the dominant Th17-associated inflammatory environment together with innate antimicrobial activation. Deconvolution largely redistributed these signals across inferred cell compartments without substantially refining the biological interpretation.

### 4.7. Limitations

Several limitations should be considered when interpreting these results. First, the modest sample size limited statistical power, particularly for per-cell type and condition-specific analyses; correlation estimates based on 5–6 samples are inherently unstable, especially when cell proportions vary within narrow ranges.

Second, the mRNA correction is a post hoc rescaling, not an independently validated cell count estimator in equine BALF, and rests on three assumptions—invariant per-cell mRNA content across activation states and disease, unbiased UMI proxies for cellular mRNA content, and multiplicative cell type-specific bias. Adjusted proportions should therefore be interpreted as improved relative abundances rather than absolute counts. Because these parameters were derived within this specific study, this heuristic requires further validation in an independent external cohort.

Third, BayesPrism requires that all relevant cell types are represented in the reference panel. Missing populations may lead to misattribution of their transcripts to the most represented cell type. For example, transcripts consistent with epithelial cells were detected in the bulk RNA-seq data but were absent from the scRNA-seq reference, leading to their misattribution to other cell types.

Fourth, DEG comparisons between deconvoluted expression profiles (Z matrix) and scRNA-seq pseudobulk profiles involve a degree of circularity, as both are derived using the same scRNA-seq reference structure. The concordance metrics reported here should therefore be interpreted as measures of internal consistency rather than external validation of cell type-specific signal recovery. An additional independent assessment would require pseudobulk data from a separate scRNA-seq cohort or RNA-seq data from flow-sorted BALF cell populations, neither of which is currently available for equine BALF.

Last limitation concerns batch, storage and sample processing effects, which were unavoidable given the study design. All bulk RNA-seq samples were processed and sequenced in a single batch, so the disease state comparison (SEA vs. CTL) within the bulk dataset was not confounded by technical batch effects. However, the scRNA-seq reference and the bulk RNA-seq datasets were generated on different platforms and library preparation chemistries. This technical variance cannot be decoupled from biological differences and likely contributes to the discrepancies observed between deconvolution-derived and scRNA-seq-derived estimates. Both scRNA-seq and bulk RNA-seq datasets were generated from cryopreserved aliquots of the same biological material, removing the fresh-vs-frozen mismatch that confounds many deconvolution validations. However, the bulk aliquots were stored for an additional ~5 years before processing. During this period, duplicate samples were stored at −80 °C, which preserves RNA integrity but may affect cell composition. Cryopreservation can reduce immune cell viability and selectively impact sensitive populations, particularly T cell subsets, and may induce stress-related transcriptional changes [51,52,53]. Previous studies reported that freezing generally preserves major immune cell populations and global gene expression patterns. However, most available studies evaluated storage durations ranging from weeks to a few months, whereas the present study involved storage periods of up to 5 years, limiting direct extrapolation to our experimental conditions [52,54,55]. Importantly, bulk RNA-seq primarily depends on preservation of RNA integrity rather than cell viability, and all samples underwent only a single freeze–thaw cycle. Moreover, no obvious depletion of T cells or upregulation of stress-related genes was observed in the present dataset. Nonetheless, subtle cryopreservation-induced compositional or transcriptional biases cannot be excluded and may have contributed to discrepancies between deconvolution and scRNA-seq-derived estimates. In addition, the same thawing, washing, and resuspension procedure used for scRNA-seq processing was intentionally applied before bulk RNA extraction to minimize technical differences between the matched aliquots. This processing strategy was selected to make the bulk and single-cell datasets as comparable as possible, rather than to optimize RNA preservation for bulk RNA-seq. We acknowledge that thawing and resuspending cells before RNA extraction is not optimal for RNA preservation, as it may allow for RNase activity, cell lysis, loss of RNA or cells in discarded supernatants, and stress-related transcriptional changes. These effects would be expected to reduce, rather than inflate, the observed agreement between bulk RNA-seq and scRNA-seq data.

Overall, subtle cryopreservation- or processing-induced compositional and transcriptional biases cannot be excluded and may have contributed to discrepancies between deconvolution and scRNA-seq-derived estimates. However, the strong agreement between bulk RNA-seq and mRNA-weighted scRNA-seq profiles suggests that these effects did not substantially compromise the main mRNA-level comparison. Better-matched prospective samples and contemporaneous processing might improve platform-level correlations but are unlikely to resolve the central limitations observed for deconvolution in this dataset, particularly mRNA content bias and limited recovery of cell type-specific differential expression. Because both datasets originated from the same biological material rather than independent cohorts, inter-dataset biological variability likely remained lower than in typical deconvolution benchmarking studies relying on external reference datasets.

## 5. Conclusions

In matched equine BALF samples, BayesPrism deconvolution accurately estimated mRNA proportions from bulk RNA-seq data compared to scRNA-seq but showed variable performance for estimating cell counts and recovering cell type-specific differential expression. Deconvolution provided limited additional biological insight beyond conventional bulk differential expression analysis, often failing to reliably attribute gene expression to specific cellular sources. In contrast, scRNA-seq unmasked distinct cell type-specific transcriptional programs underlying SEA, including Th17-associated signaling in T cells, NETosis-related activation in neutrophils, and chemokine-mediated recruitment pathways in MoMa. Notably, several biologically relevant genes were not detected in deconvoluted bulk data, likely due to dilution of low-abundance transcripts within heterogeneous cell populations.

Because the scRNA-seq reference and bulk RNA-seq data were derived from the same cohort of animals and the same biological material, deconvolution was evaluated under favorable conditions with minimal biological variability. The modest performance observed here therefore raises questions about the reliability of the deconvolution approach when applied to bulk datasets using external reference atlases, where batch effects and inter-cohort variability are expected to further reduce accuracy. Future studies should validate these findings using contemporaneous matched datasets and additional tissue types.

These findings highlight the limitations of bulk-based approaches in this dataset for resolving complex immune responses and demonstrate the unique value of scRNA-seq for identifying cell type-specific disease mechanisms, enabling biomarker discovery and therapeutic target identification in equine asthma.

## Figures and Tables

**Table 1:** Deconvolution accuracy across cell types: raw, mRNA-corrected, and mRNA-weighted comparisons. Pearson correlation (r), Spearman rank correlation (ρ), and mean absolute error (MAE) between deconvolution estimates and scRNA-seq proportions under three approaches: (1) raw BayesPrism estimates vs. cell count proportions; (2) mRNA-corrected BayesPrism estimates vs. cell count proportions; (3) raw BayesPrism estimates vs. mRNA-weighted scRNA-seq proportions. The “Overall” row represents metrics across all samples and cell types combined. Abbreviations: MAE—mean absolute error; MoMa—Monocytes/Macrophages; scRNA-seq—single-cell mRNA sequencing.

Cell Type	Raw Estimates vs. Cell Counts			mRNA-Corrected Estimates vs. Cell Counts			Raw Estimates vs. mRNA-Weighted scRNA-Seq		
	r	ρ	MAE	r	ρ	MAE	r	ρ	MAE
B cells	0.87	0.82	0.012	0.90	0.88	0.012	0.95	0.82	0.006
Dendritic cells	0.90	0.47	0.009	0.90	0.56	0.010	0.79	0.64	0.014
Mast cells	0.65	0.66	0.012	0.67	0.71	0.007	0.66	0.63	0.004
MoMa	0.11	0.27	0.398	0.06	0.32	0.129	0.53	0.36	0.076
Neutrophils	0.15	0.40	0.058	0.19	0.26	0.085	0.19	0.45	0.017
T cells	−0.09	−0.05	0.311	−0.07	−0.27	0.147	0.44	0.36	0.078
Overall	0.62	0.88	0.133	0.83	0.91	0.065	0.98	0.79	0.032

**Table 2:** Overall deconvolution performance by condition. Pearson correlation (r) and Spearman correlation (ρ) for BayesPrism estimates compared against three measures. Abbreviations: CTL—control; SEA—severe equine asthma; scRNA—single-cell mRNA sequencing.

Condition	Raw Estimates vs. Cell Counts r (ρ)	mRNA-Corrected Estimates vs. Cell Counts r (ρ)	Raw Estimates vs. mRNA-Weighted scRNA-Seq r (ρ)
CTL	0.62 (0.86)	0.77 (0.88)	0.98 (0.77)
SEA	0.63 (0.89)	0.91 (0.93)	0.99 (0.82)

**Table 3:** Comparison of differentially expressed genes between experimental scRNA-seq and BayesPrism deconvoluted expression across cell types (adjusted *p*-value < 0.05 and |log2FC| > 1). Abbreviations: DEGs—differentially expressed genes; MoMa—Monocytes/Macrophages; scRNA—single-cell mRNA sequencing.

Cell Type	scRNA-Seq DEGs	Deconvolution DEGs	Overlap
B cells	57	0	0
Dendritic cells	0	0	0
Mast cells	1	7	0
MoMa	33	93	18
Neutrophils	1195	19	5
T cells	106	84	24

**Table 4:** Concordance of differential gene expression results obtained with DESeq2 from experimental scRNA-seq and BayesPrism deconvolution across cell types. Abbreviations: log2FC—log2 fold change; MoMa—Monocytes/Macrophages.

Cell Type	Log2FC Pearson r	Log2FC Spearman ρ	Direction Concordance
Dendritic cells	−0.020	0.000	0%
Mast cells	0.128	0.079	100%
MoMa	0.456	0.409	96%
Neutrophils	0.072	−0.029	51%
T cells	0.384	0.300	94%

**Table 5:** Summary of BayesPrism deconvolution DEGs and overlap with bulk RNA-seq analysis. For each cell type, “Overlap with bulk” indicates the percentage of deconvolution-derived DEGs that were also present in the overall bulk RNA-seq DEGs list. Abbreviations: DEGs—differentially expressed genes; MoMa—Monocytes/Macrophages; RNA-seq—RNA sequencing.

Cell Type	Deconvolution DEGs	Deconvolution Only	Overlap with Bulk
B cells	0	—	—
DCs	0	—	—
Mast cells	7	1	85.7%
MoMa	93	14	84.9%
Neutrophils	19	1	94.7%
T cells	84	19	77.4%

## Data Availability

The datasets generated for this study are available in the European Nucleotide Archive (ENA) repository https://www.ebi.ac.uk, under the accession number PRJEB110841 (last updated 6 April 2026). The R code used for data analysis can be found at https://github.com/vetsuisse-unibe/equine_BALF_deconvolution (accessed on 1 May 2025).

## Supplementary Materials

The following supporting information can be downloaded at: https://www.mdpi.com/article/10.3390/genes17070773/s1, Figure S1. Quality assessment of bulk RNA-seq libraries. Figure S2. Cell count versus mRNA-weighted composition per sample. Figure S3. Condition-specific deconvolution accuracy by cell type. Figure S4. Comparison of CIBERSORTx and BayesPrism deconvolution estimates with experimental scRNA-seq data. Table S1. Per-cell type deconvolution performance by condition. Table S2. Mean cell type composition by condition across methods. Table S3. Marker genes selected per cell type. Table S4. Per-cell type and overall correlations with 95% confidence intervals. Table S5. Per-condition correlations with 95% confidence intervals. Table S6. Sensitivity of the mRNA content adjustment.

## References

[1] Couëtil L.L.L., Cardwell J.M.M., Gerber V., Lavoie J.-P.P., Léguillette R., Richard E.A.A. (2016). Inflammatory Airway Disease of Horses-Revised Consensus Statement. J. Vet. Intern. Med..

[2] Trapnell C. (2015). Defining Cell Types and States with Single-Cell Genomics. Genome Res..

[3] Sage S.E., Leeb T., Jagannathan V., Gerber V. (2024). Single-cell Profiling of Bronchoalveolar Cells Reveals a Th17 Signature in Neutrophilic Severe Equine Asthma. Immunology.

[4] Riihimäki M., Fegraeus K., Nordlund J., Waern I., Wernersson S., Akula S., Hellman L., Raine A. (2023). Single-Cell Transcriptomics Delineates the Immune Cell Landscape in Equine Lower Airways and Reveals Upregulation of FKBP5 in Horses with Asthma. Sci. Rep..

[5] Luca B.A., Steen C.B., Matusiak M., Azizi A., Varma S., Zhu C., Przybyl J., Espín-Pérez A., Diehn M., Alizadeh A.A. (2021). Atlas of Clinically Distinct Cell States and Ecosystems across Human Solid Tumors. Cell.

[6] Hippen A.A., Omran D.K., Weber L.M., Jung E., Drapkin R., Doherty J.A., Hicks S.C., Greene C.S. (2023). Performance of Computational Algorithms to Deconvolve Heterogeneous Bulk Ovarian Tumor Tissue Depends on Experimental Factors. Genome Biol..

[7] Im Y., Kim Y. (2023). A Comprehensive Overview of RNA Deconvolution Methods and Their Application. Mol. Cells.

[8] Chu T., Wang Z., Pe’er D., Danko C.G. (2022). Cell Type and Gene Expression Deconvolution with BayesPrism Enables Bayesian Integrative Analysis across Bulk and Single-Cell RNA Sequencing in Oncology. Nat. Cancer.

[9] Conning-Rowland M., Cheng C.W., Brown O., Giannoudi M., Levelt E., Roberts L.D., Griffin K.J., Cubbon R.M. (2025). Application of CIBERSORTx and BayesPrism to Deconvolution of Bulk RNA-Seq Data from Human Myocardium and Skeletal Muscle. Heliyon.

[10] QIAGEN (2023). RNeasy Mini Handbook.

[11] Tecan Group Ltd. (2025). Revelo mRNA-Seq for MagicPrep NGS User Guide, v2.

[12] Dobin A., Davis C.A., Schlesinger F., Drenkow J., Zaleski C., Jha S., Batut P., Chaisson M., Gingeras T.R. (2013). STAR: Ultrafast Universal RNA-Seq Aligner. Bioinformatics.

[13] Liao Y., Smyth G.K., Shi W. (2014). featureCounts: An Efficient General Purpose Program for Assigning Sequence Reads to Genomic Features. Bioinformatics.

[14] Love M.I., Huber W., Anders S. (2014). Moderated Estimation of Fold Change and Dispersion for RNA-Seq Data with DESeq2. Genome Biol..

[15] Satija R., Farrell J.A., Gennert D., Schier A.F., Regev A. (2015). Spatial Reconstruction of Single-Cell Gene Expression Data. Nat. Biotechnol..

[16] Racle J., de Jonge K., Baumgaertner P., Speiser D.E., Gfeller D. (2017). Simultaneous Enumeration of Cancer and Immune Cell Types from Bulk Tumor Gene Expression Data. eLife.

[17] Finotello F., Mayer C., Plattner C., Laschober G., Rieder D., Hackl H., Krogsdam A., Loncova Z., Posch W., Wilflingseder D. (2019). Molecular and Pharmacological Modulators of the Tumor Immune Contexture Revealed by Deconvolution of RNA-Seq Data. Genome Med..

[18] Zaitsev K., Bambouskova M., Swain A., Artyomov M.N. (2019). Complete Deconvolution of Cellular Mixtures Based on Linearity of Transcriptional Signatures. Nat. Commun..

[19] Bonett D.G., Wright T.A. (2000). Sample Size Requirements for Estimating Pearson, Kendall and Spearman Correlations. Psychometrika.

[20] Newman A.M., Steen C.B., Liu C.L., Gentles A.J., Chaudhuri A.A., Scherer F., Khodadoust M.S., Esfahani M.S., Luca B.A., Steiner D. (2019). Determining Cell Type Abundance and Expression from Bulk Tissues with Digital Cytometry. Nat. Biotechnol..

[21] He L., Davila-Velderrain J., Sumida T.S., Hafler D.A., Kellis M., Kulminski A.M. (2021). NEBULA Is a Fast Negative Binomial Mixed Model for Differential or Co-Expression Analysis of Large-Scale Multi-Subject Single-Cell Data. Commun. Biol..

[22] Gressler A.E., Lübke S., Wagner B., Arnold C., Lohmann K.L., Schnabel C.L. (2022). Comprehensive Flow Cytometric Characterization of Bronchoalveolar Lavage Cells Indicates Comparable Phenotypes Between Asthmatic and Healthy Horses But Functional Lymphocyte Differences. Front. Immunol..

[23] Alfei F., Kanev K., Hofmann M., Wu M., Ghoneim H.E., Roelli P., Utzschneider D.T., von Hoesslin M., Cullen J.G., Fan Y. (2019). TOX Reinforces the Phenotype and Longevity of Exhausted T Cells in Chronic Viral Infection. Nature.

[24] Dong C., Juedes A.E., Temann U.-A., Shresta S., Allison J.P., Ruddle N.H., Flavell R.A. (2001). ICOS Co-Stimulatory Receptor Is Essential for T-Cell Activation and Function. Nature.

[25] Wing J.B., Ise W., Kurosaki T., Sakaguchi S. (2014). Regulatory T Cells Control Antigen-Specific Expansion of Tfh Cell Number and Humoral Immune Responses via the Coreceptor CTLA-4. Immunity.

[26] Zotos D., Coquet J.M., Zhang Y., Light A., D’Costa K., Kallies A., Corcoran L.M., Godfrey D.I., Toellner K.-M., Smyth M.J. (2010). IL-21 Regulates Germinal Center B Cell Differentiation and Proliferation through a B Cell–Intrinsic Mechanism. J. Exp. Med..

[27] Joshi S., Pantalena L.-C., Liu X.K., Gaffen S.L., Liu H., Rohowsky-Kochan C., Ichiyama K., Yoshimura A., Steinman L., Christakos S. (2011). 1,25-Dihydroxyvitamin D 3 Ameliorates Th17 Autoimmunity via Transcriptional Modulation of Interleukin-17A. Mol. Cell. Biol..

[28] Tatler A.L., John A.E., Jolly L., Habgood A., Porte J., Brightling C., Knox A.J., Pang L., Sheppard D., Huang X. (2011). Integrin Avβ5-Mediated TGF-β Activation by Airway Smooth Muscle Cells in Asthma. J. Immunol..

[29] de la Fuente H., Cruz-Adalia A., Martinez del Hoyo G., Cibrián-Vera D., Bonay P., Pérez-Hernández D., Vázquez J., Navarro P., Gutierrez-Gallego R., Ramirez-Huesca M. (2014). The Leukocyte Activation Receptor CD69 Controls T Cell Differentiation through Its Interaction with Galectin-1. Mol. Cell. Biol..

[30] Miyazaki H., Wannakul T., Yang S., Yang D., Karasawa A., Shishido A., Cao R., Yamamoto Y., Kagawa Y., Kobayashi S. (2025). FABP7 in Hepatic Macrophages Promotes Fibroblast Activation and CD4+ T-Cell Migration by Regulating M2 Polarization During Liver Fibrosis. J. Immunol. Res..

[31] Shimizu Y., Dobashi K. (2012). CC-Chemokine CCL15 Expression and Possible Implications for the Pathogenesis of IgE-Related Severe Asthma. Mediat. Inflamm..

[32] Prikk K., Maisi P., Pirilä E., Reintam M.-A., Salo T., Sorsa T., Sepper R. (2002). Airway Obstruction Correlates with Collagenase-2 (MMP-8) Expression and Activation in Bronchial Asthma. Lab. Investig..

[33] Skerlavaj B., Scocchi M., Gennaro R., Risso A., Zanetti M. (2001). Structural and Functional Analysis of Horse Cathelicidin Peptides. Antimicrob. Agents Chemother..

[34] Han Y., Ji X., Rao J., Zhang Y., Chen X., Gong F. (2026). TNFAIP3, PADI4, and CXCR4 as Biomarkers of Neutrophil Extracellular Traps in Severe Asthma. Int. Immunopharmacol..

[35] Jiang S., Park D.W., Tadie J.-M., Gregoire M., Deshane J., Pittet J.F., Abraham E., Zmijewski J.W. (2014). Human Resistin Promotes Neutrophil Proinflammatory Activation and Neutrophil Extracellular Trap Formation and Increases Severity of Acute Lung Injury. J. Immunol..

[36] Quoc Q.L., Choi Y., Thi Bich T.C., Yang E.-M., Shin Y.S., Park H.-S. (2021). S100A9 in Adult Asthmatic Patients: A Biomarker for Neutrophilic Asthma. Exp. Mol. Med..

[37] Sprenkeler E.G.G., Zandstra J., van Kleef N.D., Goetschalckx I., Verstegen B., Aarts C.E.M., Janssen H., Tool A.T.J., van Mierlo G., van Bruggen R. (2022). S100A8/A9 Is a Marker for the Release of Neutrophil Extracellular Traps and Induces Neutrophil Activation. Cells.

[38] Alevy Y.G., Patel A.C., Romero A.G., Patel D.A., Tucker J., Roswit W.T., Miller C.A., Heier R.F., Byers D.E., Brett T.J. (2012). IL-13–Induced Airway Mucus Production Is Attenuated by MAPK13 Inhibition. J. Clin. Investig..

[39] Cremades-Jimeno L., de Pedro M.Á., López-Ramos M., Sastre J., Mínguez P., Fernández I.M., Baos S., Cárdaba B. (2021). Prioritizing Molecular Biomarkers in Asthma and Respiratory Allergy Using Systems Biology. Front. Immunol..

[40] Liu L., Zhang X., Liu Y., Zhang L., Zheng J., Wang J., Hansbro P.M., Wang L., Wang G., Hsu A.C.Y. (2019). Chitinase-like Protein YKL-40 Correlates with Inflammatory Phenotypes, Anti-Asthma Responsiveness and Future Exacerbations. Respir. Res..

[41] Anguita E., Candel F.J., Chaparro A., Roldán-Etcheverry J.J. (2017). Transcription Factor GFI1B in Health and Disease. Front. Oncol..

[42] Taketomi Y., Sugiki T., Saito T., Ishii S.-I., Hisada M., Suzuki-Nishimura T., Uchida M.K., Moon T.-C., Chang H.-W., Natori Y. (2003). Identification of NDRG1 as an Early Inducible Gene during In Vitro Maturation of Cultured Mast Cells. Biochem. Biophys. Res. Commun..

[43] Velotti F., Barchetta I., Cimini F.A., Cavallo M.G. (2020). Granzyme B in Inflammatory Diseases: Apoptosis, Inflammation, Extracellular Matrix Remodeling, Epithelial-to-Mesenchymal Transition and Fibrosis. Front. Immunol..

[44] Monaco G., Lee B., Xu W., Mustafah S., Hwang Y.Y., Carré C., Burdin N., Visan L., Ceccarelli M., Poidinger M. (2019). RNA-Seq Signatures Normalized by MRNA Abundance Allow Absolute Deconvolution of Human Immune Cell Types. Cell Rep..

[45] Maden S.K., Kwon S.H., Huuki-Myers L.A., Collado-Torres L., Hicks S.C., Maynard K.R. (2023). Challenges and Opportunities to Computationally Deconvolve Heterogeneous Tissue with Varying Cell Sizes Using Single-Cell RNA-Sequencing Datasets. Genome Biol..

[46] Lu S., Yang J., Yan L., Liu J., Wang J.J., Jain R., Yu J. (2025). Transcriptome Size Matters for Single-Cell RNA-Seq Normalization and Bulk Deconvolution. Nat. Commun..

[47] Grieshaber-Bouyer R., Radtke F.A., Cunin P., Stifano G., Levescot A., Vijaykumar B., Nelson-Maney N., Blaustein R.B., Monach P.A., Nigrovic P.A. (2021). The Neutrotime Transcriptional Signature Defines a Single Continuum of Neutrophils across Biological Compartments. Nat. Commun..

[48] Avila Cobos F., Alquicira-Hernandez J., Powell J.E., Mestdagh P., De Preter K. (2020). Benchmarking of Cell Type Deconvolution Pipelines for Transcriptomics Data. Nat. Commun..

[49] Dietrich A., Merotto L., Pelz K., Eder B., Zackl C., Reinisch K., Edenhofer F., Marini F., Sturm G., List M. (2026). Omnideconv: A Unifying Framework for Using and Benchmarking Single-Cell-Informed Deconvolution of Bulk RNA-Seq Data. Genome Biol..

[50] Meng G., Tang W., Huang E., Li Z., Feng H. (2023). A Comprehensive Assessment of Cell Type-Specific Differential Expression Methods in Bulk Data. Brief. Bioinform..

[51] Madissoon E., Wilbrey-Clark A., Miragaia R.J., Saeb-Parsy K., Mahbubani K.T., Georgakopoulos N., Harding P., Polanski K., Huang N., Nowicki-Osuch K. (2020). ScRNA-Seq Assessment of the Human Lung, Spleen, and Esophagus Tissue Stability after Cold Preservation. Genome Biol..

[52] Guillaumet-Adkins A., Rodríguez-Esteban G., Mereu E., Mendez-Lago M., Jaitin D.A., Villanueva A., Vidal A., Martinez-Marti A., Felip E., Vivancos A. (2017). Single-Cell Transcriptome Conservation in Cryopreserved Cells and Tissues. Genome Biol..

[53] Denisenko E., Guo B.B., Jones M., Hou R., de Kock L., Lassmann T., Poppe D., Clément O., Simmons R.K., Lister R. (2020). Systematic Assessment of Tissue Dissociation and Storage Biases in Single-Cell and Single-Nucleus RNA-Seq Workflows. Genome Biol..

[54] Wohnhaas C.T., Leparc G.G., Fernandez-Albert F., Kind D., Gantner F., Viollet C., Hildebrandt T., Baum P. (2019). DMSO Cryopreservation Is the Method of Choice to Preserve Cells for Droplet-Based Single-Cell RNA Sequencing. Sci. Rep..

[55] Browne D.J., Miller C.M., Doolan D.L. (2024). Technical Pitfalls When Collecting, Cryopreserving, Thawing, and Stimulating Human T-Cells. Front. Immunol..

