# Supplementary material for: Cell-Type Deconvolution of Equine BALF RNA-Seq: A Critical Comparison with Matched Single-Cell Data
Source: Genes (Basel). 2026 Jun 30;17(7):773. doi: 10.3390/genes17070773 (PMC13408615; doi:10.3390/genes17070773)
Supplement: Supplementary file 1 [file genes-17-00773-s001.zip › genes-4292059-supplementary.pdf]

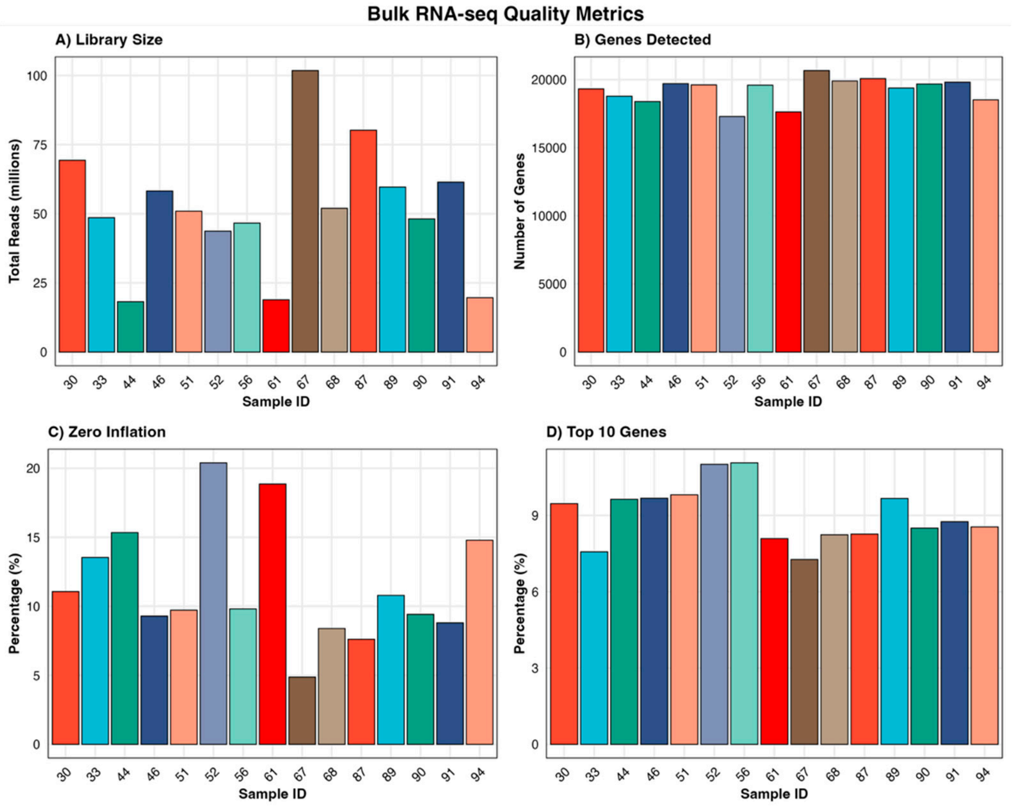

Figure S1. Quality assessment of bulk RNA-seq libraries. Four quality metrics across 15 BALF bulk RNA-seq samples. (**A**) Library size, shown as total reads in millions. (**B**) Number of genes detected per sample. (**C**) Zero inflation, defined as the percentage of genes with zero counts. (**D**) Concentration of expression, measured as the cumulative percentage of total counts attributed to the ten most highly expressed genes. Sample IDs are shown on the x-axis.

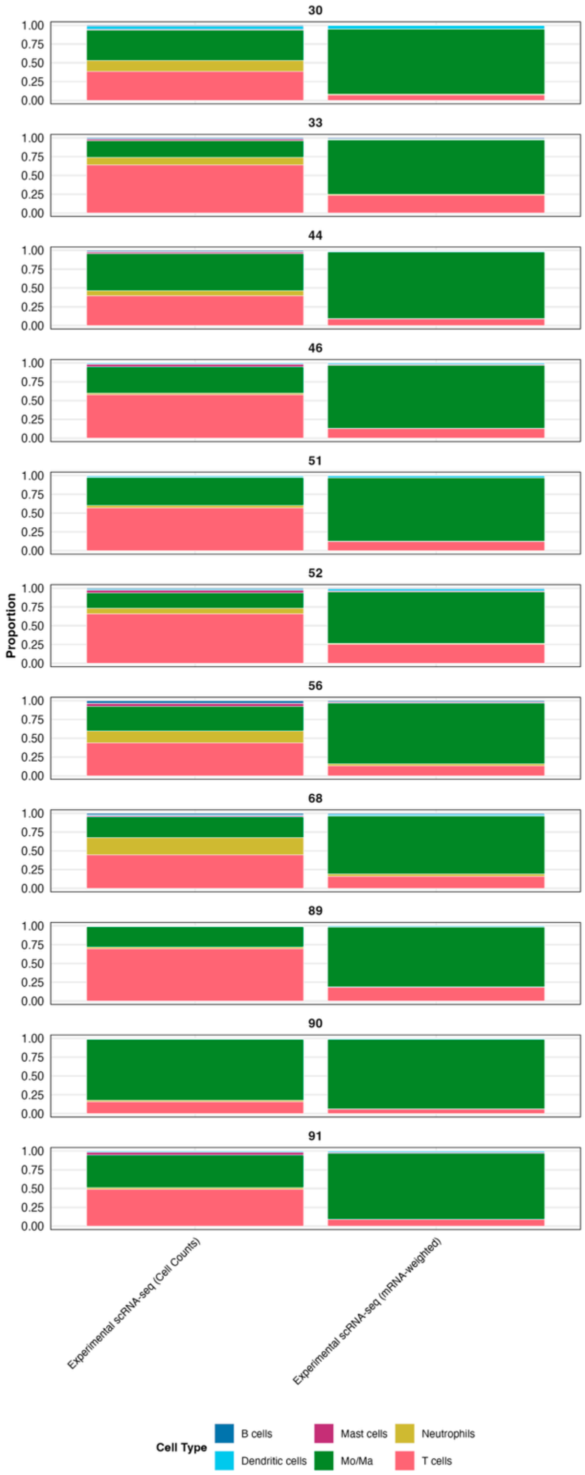

Figure S2. Cell count versus mRNA-weighted composition per sample. Stacked bar plots comparing cell-type proportions derived from experimental scRNA-seq using two approaches for each of the 11 matched BALF samples. Left bars show cell count-based proportions (number of cells per type divided by total cells), and right bars show mRNA-weighted proportions (total UMI counts per cell type divided by total library size). MoMa dominate the mRNA-weighted composition (69–92%) due to their high per-cell mRNA content, whereas cell count-based proportions show a more balanced distribution with substantial T cell fractions. *Abbreviations: Mo/Ma, Monocytes/Macrophages; scRNAseq, single-cell mRNA sequencing.*

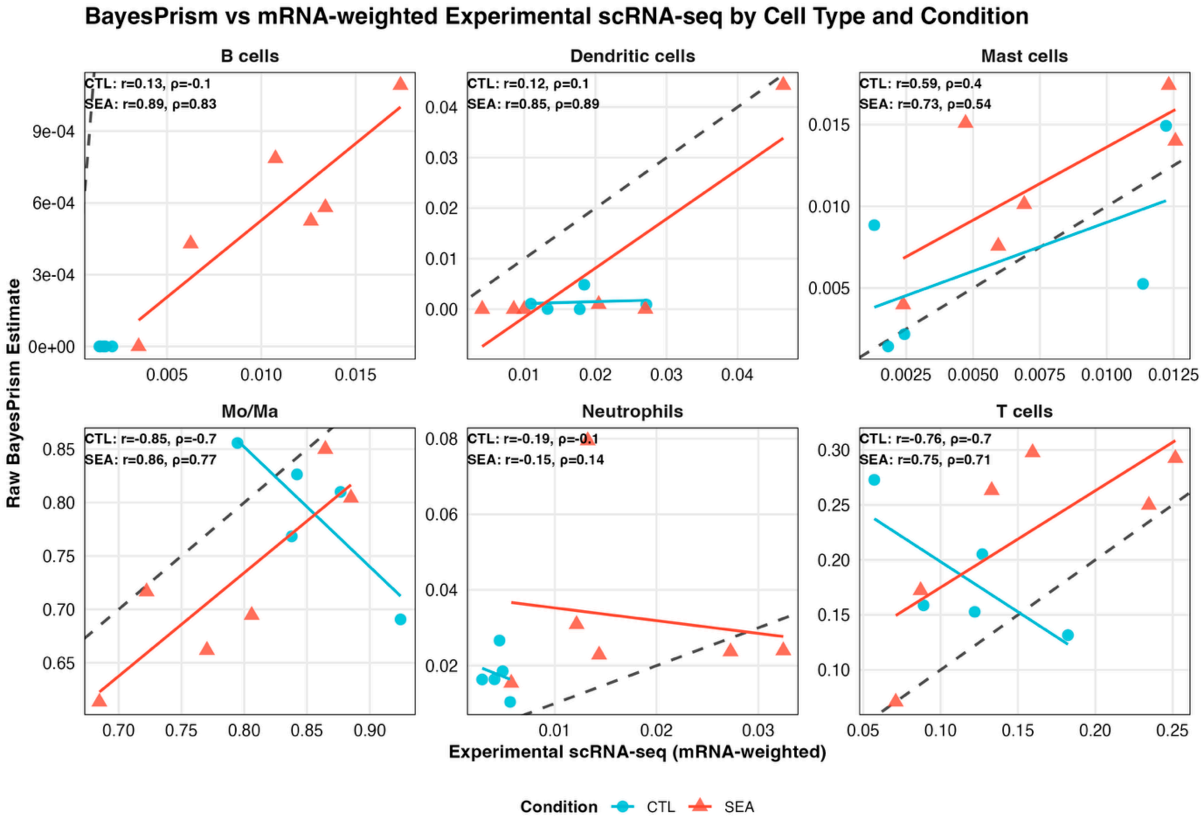

Figure S3. Condition-specific deconvolution accuracy by cell type. Scatter plots comparing raw BayesPrism estimates against mRNA-weighted experimental scRNA-seq proportions for each of the six major cell types, stratified by condition (CTL, blue circles; SEA, red triangles). Regression lines are shown per condition. Pearson correlation (r) and Spearman rank correlation (ρ) are displayed for each condition within each panel. The dashed diagonal line indicates perfect agreement. SEA samples generally showed stronger concordance than CTL samples, particularly for B cells, dendritic cells, MoMa, and T cells. Notably, MoMa and T cells exhibited negative correlations in CTL, likely reflecting the limited dynamic range of these populations across control samples. *Abbreviations: CTL, control; SEA, severe equine asthma; Mo/Ma, Monocytes/Macrophages*

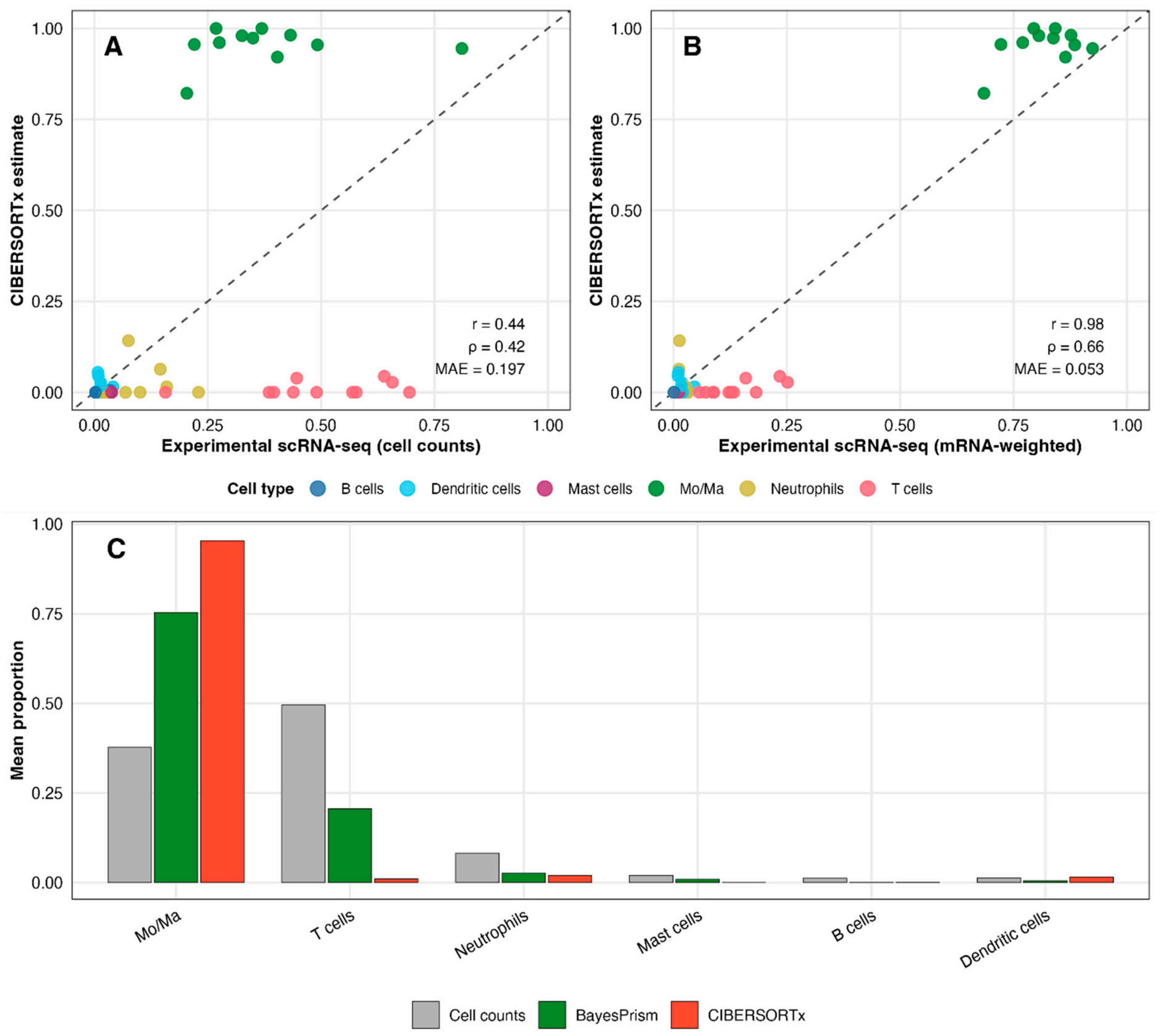

Figure S4. Comparison of CIBERSORTx and BayesPrism deconvolution estimates with experimental scRNA-seq data. Scatter plots comparing CIBERSORTx-estimated cell type proportions with experimental scRNA-seq-derived proportions for matched samples. (**A**) Raw CIBERSORTx estimates vs. experimental scRNA-seq cell count proportions, showing systematic overestimation of MoMa and underestimation of T cells. (**B**) Raw CIBERSORTx estimates vs. mRNA-weighted experimental scRNA-seq proportions, confirming that deconvolution accurately captures mRNA-derived proportions. Points are colored by cell type; dashed line indicates perfect agreement (y = x). (**C**) Mean estimated proportion per cell type across experimental scRNA-seq cell counts, BayesPrism, and CIBERSORTx, demonstrating that both algorithmic frameworks systematically overestimate the high-mRNA monocyte/macrophage compartment and underestimate the low-mRNA T-cell compartment. *Abbreviations: CTL, control; SEA, severe equine asthma; Mo/Ma, Monocytes/Macrophages*

Table S1. Per-cell type deconvolution performance by condition. Pearson correlation (r) with Spearman rank correlation (ρ) in parentheses for BayesPrism estimates compared against: (1) Original cell count proportions; (2) Corrected cell count proportions after mRNA content adjustment; and (3) mRNA-weighted experimental scRNA-seq proportions. *Abbreviations: CTL, control; SEA, severe equine asthma; scRNA, single-cell mRNA sequencing.*

Cell type	Original r (ρ)		Corrected r (ρ)		mRNA-weighted experimental scRNA-seq r (ρ)	
	CTL	SEA	CTL	SEA	CTL	SEA
B cells	0.53 (0.80)	0.69 (0.26)	0.67 (0.90)	0.74 (0.31)	0.13 (- 0.10)	0.89 (0.83)
Dendritic cells	0.30 (- 0.10)	0.94 (0.89)	0.33 (0.20)	0.94 (0.89)	0.12 (0.10)	0.85 (0.89)
Mast cells	0.60 (0.30)	0.70 (0.66)	0.68 (0.30)	0.59 (0.66)	0.59 (0.40)	0.73 (0.54)
MoMa	- 0.90 (- 0.70)	0.82 (0.77)	- 0.87 (- 0.70)	0.77 (0.77)	- 0.85 (- 0.70)	0.86 (0.77)
Neutrophils	- 0.22 (- 0.40)	- 0.33 (0.26)	0.26 (- 0.30)	- 0.29 (- 0.09)	- 0.19 (- 0.10)	- 0.15 (0.14)
T cells	- 0.89 (- 0.70)	0.58 (0.71)	- 0.78 (- 0.70)	0.33 (0.26)	- 0.76 (- 0.70)	0.75 (0.71)

Table S2. Mean cell type composition by condition across methods. Cell types were merged to match cytology classification: MoMa/DC = Monocytes/Macrophages + Dendritic cells; Lymphocytes = T cells + B cells. *Abbreviations: BP, BayesPrism; Cytology Pre, cytology before BALF processing; Cytology Post, cytology after BALF processing (before freezing); scRNA-seq, single-cell mRNA sequencing CTL, control; SEA, severe equine asthma.*

Condition	Method	MoMa/DC (%)	Lymphocytes (%)	Neutrophils (%)	Mast cells (%)
CTL	scRNA-seq	45.8	50.1	2.4	1.7
	Cytology Pre	53.2	41.0	4.9	1.0
	Cytology Post	50.8	44.7	1.5	2.9
	BP Original	79.2	18.4	1.8	0.7
	BP Corrected	31.0	56.8	10.6	1.6
SEA	scRNA-seq	33.4	51.3	13.0	2.3
	Cytology Pre	45.7	33.0	19.5	1.8
	Cytology Post	50.2	37.1	9.8	2.8
	BP Original	73.1	22.5	3.3	1.1
	BP Corrected	25.3	56.3	16.2	2.2

Table S3. Marker genes selected per cell type. Cell type-specific marker genes were identified using BayesPrism's get.exp.stat function (pseudo-count = 0.1, minimum cell count = 10) and cell type-specific marker genes were selected using select.marker (*P*-value < 0.01, log2 fold change (log2FC) > 0.1). *Abbreviations: MoMa, monocytes/macrophages.*

Cell type	Unique markers
B cells	636
Dendritic cells	1,033
Mast cells	381
MoMa	669
Neutrophils	303
T cells	452
**Total**	**3,474**

Table S4. **Per-cell-type and overall correlations with 95% confidence intervals.** Pearson and Spearman rank correlations between BayesPrism estimates and scRNA-seq–derived proportions, with 95% confidence intervals, for the three benchmarking comparisons summarized in Table 1. Confidence intervals were computed using Fisher's z-transformation, with the Bonett & Wright (2000) standard-error correction applied to Spearman correlations. n indicates the number of paired observations contributing to each correlation. *Abbreviations: CI, confidence interval; MoMa, monocytes/macrophages.*

A. Raw BayesPrism estimates (θ) vs. scRNA-seq cell-count proportions

Cell type	Pearson *r* (95% CI)	Spearman ρ (95% CI)
B cells	0.873 (0.574, 0.967)	0.818 (0.337, 0.960)
Dendritic cells	0.897 (0.645, 0.973)	0.473 (−0.214, 0.847)
Mast cells	0.654 (0.088, 0.900)	0.664 (0.034, 0.916)
MoMa	0.105 (−0.528, 0.663)	0.273 (−0.402, 0.755)
Neutrophils	0.146 (−0.497, 0.686)	0.400 (−0.288, 0.816)
T cells	−0.090 (−0.655, 0.539)	−0.045 (−0.628, 0.570)
**Overall**	**0.622 (0.448, 0.751)**	**0.884 (0.802, 0.934)**

B. mRNA-adjusted BayesPrism estimates vs. scRNA-seq cell-count proportions

Cell type	Pearson *r* (95% CI)	Spearman ρ (95% CI)
B cells	0.899 (0.649, 0.974)	0.882 (0.513, 0.976)
Dendritic cells	0.895 (0.638, 0.973)	0.564 (−0.107, 0.882)
Mast cells	0.672 (0.121, 0.906)	0.709 (0.110, 0.930)
MoMa	0.062 (−0.559, 0.638)	0.318 (−0.363, 0.778)
Neutrophils	0.185 (−0.467, 0.707)	0.264 (−0.409, 0.751)
T cells	−0.069 (−0.642, 0.554)	−0.273 (−0.755, 0.402)
**Overall**	**0.833 (0.740, 0.894)**	**0.908 (0.841, 0.948)**

C. Raw BayesPrism estimates (θ) vs. mRNA-weighted scRNA-seq proportions

**Cell type**	**Pearson *r* (95% CI)**	**Spearman ρ (95% CI)**
B cells	0.953 (0.824, 0.988)	0.818 (0.337, 0.960)
Dendritic cells	0.792 (0.367, 0.944)	0.636 (−0.008, 0.907)
Mast cells	0.657 (0.094, 0.901)	0.627 (−0.021, 0.904)
MoMa	0.528 (−0.105, 0.857)	0.355 (−0.331, 0.795)
Neutrophils	0.194 (−0.459, 0.711)	0.445 (−0.243, 0.835)
T cells	0.439 (−0.218, 0.822)	0.355 (−0.331, 0.795)
**Overall**	**0.983 (0.972, 0.989)**	**0.792 (0.661, 0.877)**

Table S5. Per-condition correlations with 95% confidence intervals. Pearson and Spearman rank correlations between BayesPrism estimates and scRNA-seq–derived proportions, computed separately for control (CTL) and severe equine asthma (SEA) samples, with 95% confidence intervals, for the three benchmarking comparisons summarized in Table 2. Confidence intervals were computed using Fisher's *z*-transformation, with the Bonett & Wright (2000) standard-error correction applied to Spearman correlations. *n* indicates the number of paired observations contributing to each correlation. *Abbreviations: CI, confidence interval; CTL, control; SEA, severe equine asthma.*

**Comparison**	**Group**	**Pearson *r* (95% CI)**	**Spearman ρ (95% CI)**
Raw BayesPrism (θ) vs. cell counts	CTL	0.623 (0.338, 0.803)	0.863 (0.698, 0.941)
Raw BayesPrism (θ) vs. cell counts	SEA	0.625 (0.373, 0.791)	0.894 (0.777, 0.951)
mRNA-adjusted vs. cell counts	CTL	0.771 (0.568, 0.885)	0.876 (0.723, 0.947)
mRNA-adjusted vs. cell counts	SEA	0.908 (0.825, 0.952)	0.933 (0.854, 0.970)
Raw BayesPrism (θ) vs. mRNA-weighted scRNA-seq	CTL	0.979 (0.955, 0.990)	0.773 (0.535, 0.897)
Raw BayesPrism (θ) vs. mRNA-weighted scRNA-seq	SEA	0.987 (0.974, 0.993)	0.823 (0.648, 0.916)

Table S6. Sensitivity of the mRNA-content adjustment. Robustness of post hoc adjustments (Methods section 5.3.1) to (A) sample-level reference variation and (B) disease-state-specific computation of m_k. Based on n = 11 matched samples (5 CTL, 6 SEA) and 60,262 reference cells (six cell types). Full-reference baseline performance (mean m_k across all samples): Pearson r = 0.833, Mean Absolute Error (MAE) = 0.065 versus true scRNA-seq proportions. *Abbreviations: CTL, control; CV, coefficient of variation; LOSO, leave-one-sample-out; MoMa, monocytes/macrophages; SEA, severe equine asthma; UMI, unique molecular identifier.*

A. Leave-one-sample-out cross-validation of the scRNA-seq reference. For each of the 11 matched samples, the cell-type-specific mean per-cell mRNA content (m_k) was recomputed from the remaining 10 samples and the adjustment was reapplied. Reported values are the range and coefficient of variation of m_k across the 11 LOSO folds.

Cell type	Full-reference m_k (UMI)	LOSO min (UMI)	LOSO max (UMI)	Range (% of full)	CV across folds (%)
B cells	3,882	3,686	4,101	10.7	2.8
Dendritic cells	11,188	10,903	11,438	4.8	1.6
Mast cells	2,492	2,342	2,562	8.8	2.4
MoMa	16,362	15,801	18,613	17.2	4.6
Neutrophils	1,010	967	1,052	8.5	2.2
T cells	1,967	1,910	2,036	6.4	2.0

B. Disease-state-specific m_k. m_k was recomputed separately using only CTL or only SEA samples and applied per sample by condition. Values are the mean per-cell UMI count for each cell type within each condition, and the SEA/CTL fold difference.

Cell type	*m*_CTL (UMI)	*m*_SEA (UMI)	SEA/CTL ratio
B cells	3,736	3,900	1.04
Dendritic cells	11,226	11,161	0.99
Mast cells	2,856	2,298	0.80
MoMa	14,108	18,772	1.33
Neutrophils	1,266	973	0.77
T cells	1,658	2,214	1.34
